# Protective Coating for Stable Cycling of Li-Metal Batteries Based on Cellulose and Single-Ion Conducting Polymer

**DOI:** 10.1021/acsami.4c13335

**Published:** 2024-11-25

**Authors:** Mariana Vargas Ordaz, Nejc Pavlin, Matteo Gastaldi, Claudio Gerbaldi, Robert Dominko

**Affiliations:** a National Institute of Chemistry, Hajdrihova 19, Ljubljana SI-1000, Slovenia; b Faculty of Chemistry and Chemical Technology, University of Ljubljana, Večna pot 113, Ljubljana SI-1001, Slovenia; c ALISTORE -European Research Institute, 33 rue Saint-Leu, Amiens 80039, Cedex, France; d GAME Lab, Department of Applied Science and Technology (DISAT), Politecnico di Torino, Corso Duca degli Abruzzi 24, Torino 10129, Italy; e National Reference Center for Electrochemical Energy Storage (GISEL) − INSTM, Via G. Giusti 9, Firenze 50121, Italy

**Keywords:** protective coating, lithium metal batteries, cellulose, single ion conductor, P(LiMTFSI)

## Abstract

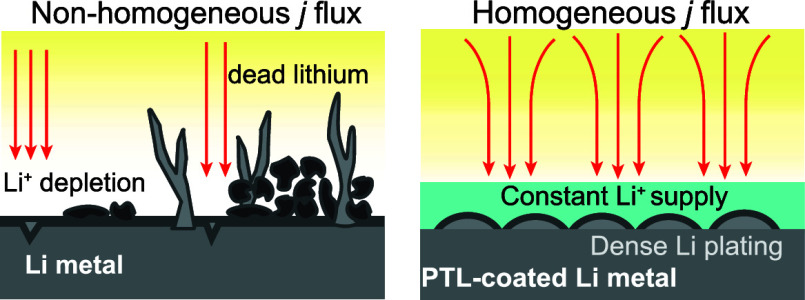

The thermodynamically unstable interface between metallic lithium and electrolyte poses a major problem for the massive commercialization of Li-metal batteries. In this study, we propose the use of a multicomponent protective coating based on cellulose modified with dimethylthexylsilyl group (TDMSC), single-ion conducting polymer P(LiMTFSI), and LiNO_3_ (TDMSC-P(LiMTFSI)-LiNO_3_, namely PTL). The coating shows its positive effect by increasing the Coulombic efficiency in Li || Cu cells from 95.9 and 98.6% for bare Li, to >99.3% for Li coated (Li@PTL), with 1 M LiFSI in FEC:DEC and 1 M LiFSI in DME electrolyte, respectively. Symmetrical Li || Li PTL-coated cells exhibit a much more prolonged and stable cycling with a slower increase in overpotential compared to bare Li cells. Li@PTL anodes enable improved cycling of Li@PTL/LFP cells compared to noncoated cells in liquid electrolytes. In this respect, inhibition of high surface area lithium growth is confirmed through postcycling scanning electron microscopy. Remarkably, dendrite-free galvanostatic cycling is demonstrated in laboratory-scale solid-state battery cells assembled with LFP composite cathode (catholyte configuration with PEO + LiTFSI as ionically conducting binder) and a cross-linked PEO-based solid polymer electrolyte. The PTL protective coating enables improved stability of Li metal batteries in combination with smooth transport of Li^+^ at the electrode–electrolyte interface and homogeneous lithium coating, highlighting its promising prospects in enhancing the performance and safety of lithium metal batteries by properly tuning the synergy between the coating components.

## 1 Introduction

Low lithium (Li) metal thermodynamic stability is one of the main bottlenecks that limits, at present, its use in high-capacity batteries. The inherent presence of a solid electrolyte interphase (SEI) on Li metal, when in contact with any liquid electrolyte, allows it to be cycled. However, due to its heterogeneous composition and lack of mechanical and chemical stability, SEI fails to provide a robust interface that can withstand the massive volumetric changes that Li metal suffers during charge/discharge.^1^ In the quest to fully exploit Li metal’s physicochemical characteristics, high specific capacity (3860 mA h g^–1^), and lowest electrochemical potential (−3.04 V vs SHE), several approaches have been proposed to improve its stability.^2^ Among others, literature reports include modifying the electrolyte composition to form a more stable SEI,^3^ 3D host current collectors,^4,5^ separator modification,^6,7^ artificial SEI,^8−11^ and protective coatings.^12−15^ The implementation of protective coatings is highlighted by the major role that the interface between Li metal and electrolyte plays in the transport of Li^+^ ions.^16^ Therefore, adequately modifying the interfacial characteristics allows for proper tuning of how the Li plating and stripping processes occur. In this sense, the presence of a protective coating can provide additional benefits, such as homogeneous transport of Li^+^, enhanced electrochemical stability, and lower reactivity to reduce current “hot spots” that increase the risk of dendrite generation and growth.^17^

The main characteristics that should be considered when designing a protective coating are uniform and moderate thickness, excellent mechanical strength and adhesion to the lithium surface, electronic insulation but ionic conduction, pinhole-free, and excellent chemical and electrochemical stability.^18^ These properties can rarely be fulfilled by one single material; therefore, multicomponent protective coatings are commonly reported.^19^ Composite coatings can benefit from the properties’ interplay among both groups of materials, helping to tune the desired effect. In this regard, polymers appear as a desirable option due to their elastic properties, flexibility, and lightweight nature, which can prevent cracking and thus maintain the coating’s integrity. Additionally, polysaccharides can offer multiple benefits in the design of battery materials due to their excellent chemical and mechanical properties, as well as their sustainable features in terms of low price and wide availability.^20^ Cellulose stands out in this respect, thanks to its film-forming ability, high abundance, and high Young modulus (20–30 GPa),^21^ which can effectively mitigate the high surface area lithium (HSAL) formation. However, the main challenge that hinders its use is the lack of processability, which is a major reason for the limited research on cellulose as the sole polymer matrix in battery materials.^22^ One way to overcome such a problem is to derivatize the cellulose chains by exchanging the free hydroxyl groups for other functionalities that can improve their processability (i.e., solubility, melting point, etc.). For instance, silicon moieties are known for their interface hardening or gelation,^23^ and silicon-containing polymers have shown excellent adhesion strength, thermal stability, and chemical inertness.^24^

Single-ion conducting polymers, which enable high Li^+^ mobility by binding their anionic component to a polymeric backbone, can promote a transference number (*t*_Li+_) close to unity and reduce the concentration gradients that occur during battery cycling.^25^ However, their application is mainly limited to solid polymer electrolytes,^26−29^ with few studies on protective coatings.^30^ Therefore, investigating their application in thin interlayers for Li metal is relevant due to the potential impact on Li^+^ transport at the interface.

Lithium nitrate (LiNO_3_) is a well-known and widely studied additive commonly used in liquid electrolytes due to its properties as a grain refiner in the early stages of Li plating. When nitrate anions (NO_3_^–^) are present, they are adsorbed on the surface of Li metal, building an electric double-layer structure that has an impact on the increase in the plating overpotential, which reduces the size of both Li nuclei and grains.^31^ However, its low solubility (<0.05 M) in carbonate-based electrolytes limits its widespread use in commercial batteries.^32^ One way to circumvent this challenge is to incorporate LiNO_3_ in a scaffold or matrix to generate a Li reservoir at the electrode.^33−35^

Herein, we report the development of a drop-casted protective coating based on cellulose (2-6-di-*O*-thexyldimethylsilylcellulose), single-ion conducting polymer P(LiMTFSI), and LiNO_3_ to inhibit HSAL formation, promote homogeneous flux of lithium, and stabilize Li metal surface upon cycling. The interlayer performance is assessed by comparing the galvanostatic behavior of coated and no-coated lithium metal in symmetrical Li || Li and asymmetrical Li || Cu configurations, in terms of stability and Coulombic efficiency. As a proof of concept, the promising features of the protective coating for future development of stable and safe Li-based batteries are evaluated in laboratory-scale Li-metal cells with LiFePO_4_ as cathode material in two configurations, with liquid or polymer-based solid electrolytes, where the role of interface stabilization as well as the protective and conductive nature of the coating during prolonged cycling are positively confirmed.

## 2 Experimental Section

### 2.1 Materials

Microcrystalline cellulose (Alfa Aesar, 99%), lithium chloride (LiCl, Sigma-Aldrich, 99%), and lithium nitrate (LiNO_3_, Merck, 99.5%) were dried in a glass vacuum oven at 100 °C for 12 h. Lithium bis(trifluoromethane)sulfonimide (LiTFSI, Solvionic, 99.0%) and poly(ethylene oxide) (PEO, *M*_n_ = 400k) were dried under reduced pressure for 24 h at 120 and 50 °C, respectively. Poly(trifluoromethane)sulfonimide lithium methacrylate (P(LiMTFSI), Specific Polymers, 99%) was dried under reduced pressure overnight. Chloro(dimethyl)thexylsilane (TDMS-Cl, Merck, 95%), imidazole (Sigma-Aldrich, 99.5%), lithium bis(fluorosulfonyl)imide (LiFSI, Solvionic, 99.9%), phosphate-buffered saline (pH 7.4, Merck), polyvinylidene fluoride (PVdF, Sigma-Aldrich), benzophenone (BP, Sigma-Aldrich, 99%), and lithium iron phosphate (LiFePO_4_/C, LFP, Targray, SLFP02002) were used as received.

*N,N*-Dimethylacetamide (DMAc, Alfa Aesar, 99%), 1,2-dimethoxyethane (DME, Merck, 99.5%), and tetrahydrofuran (THF, Honey Well, ≥ 99.9%) were dried with 4 Å molecular sieves for at least 5 days, refluxed with Na/K alloy (ca. 1 mL l^–1^) overnight, and then fractionally distilled. The final water content in distilled solvents was below 1 ppm, as determined by Karl Fischer titration. Fluoroethylene carbonate (FEC, Alfa Aesar, 98%), anhydrous diethyl carbonate (DEC, Sigma-Aldrich, 99%), and *N*-methyl-2-pyrrolidone (NMP, Merck) were used without any further treatment. Deuterated chloroform (CDCl_3_, 99.8 atom %D, w/o TMS, stabilized w/Ag, Armar Isotopes) reference peak at 7.26 ppm was used as a solvent during NMR analysis.

### 2.2 Synthesis of TDMSC Cellulose Material

**Figure 1:**
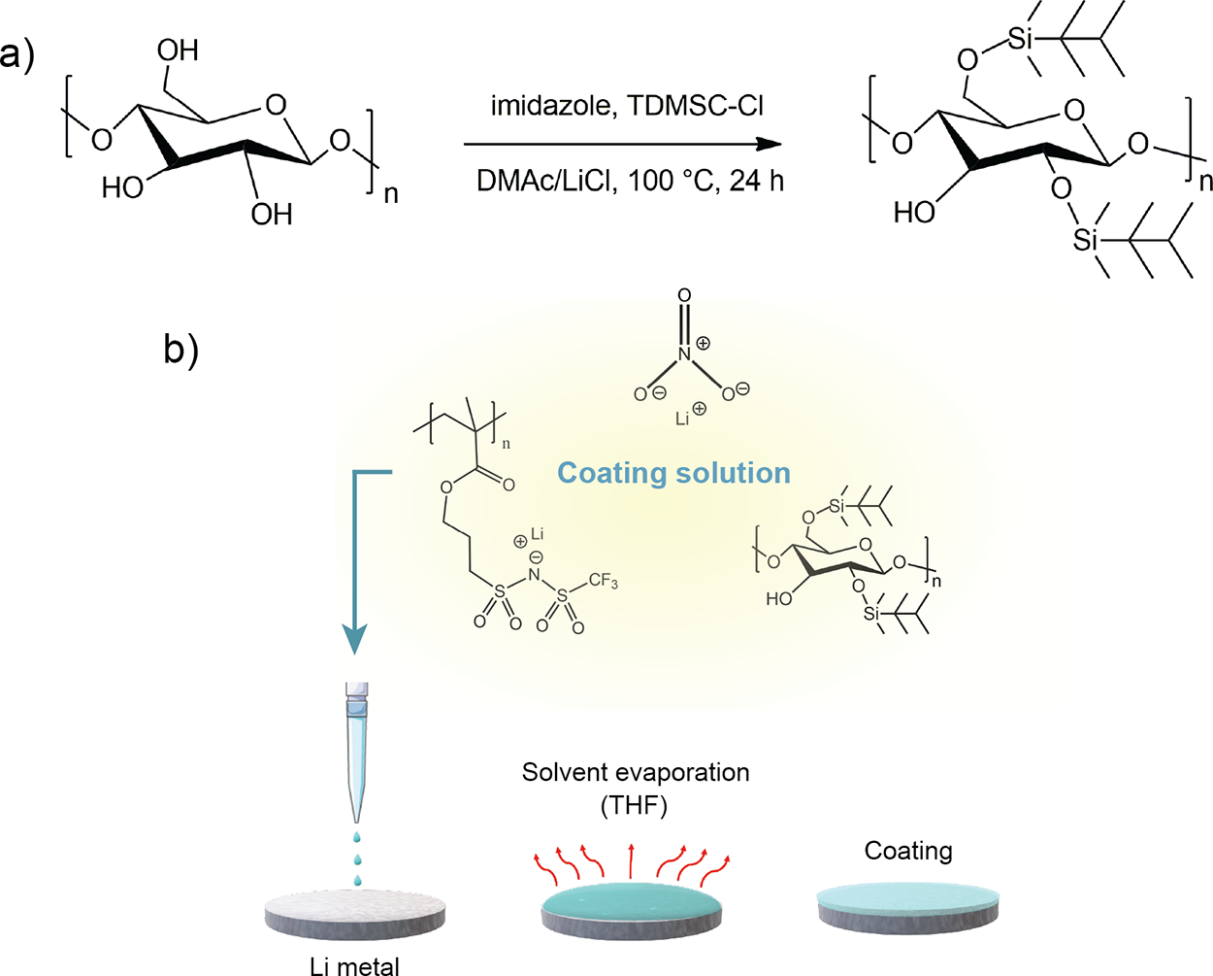
(a) Synthesis conditions to obtain 2-6-di-*O*-thexyldimethylsilylcellulose from microcrystalline cellulose, and (b) drop casting procedure employed to produce protective coating for Li metal.

2–6-Di-*O*-thexyldimethylsilylcellulose (TDMSC) was synthesized according to a previously reported method^36^ (sketched in Figure 1a), with some modifications. Microcrystalline cellulose was dissolved by suspending 5 g (30.8 mmol) in 125 mL of DMAc at 120 °C. After 2 h, the temperature was decreased to 100 °C, and 7.5 g (176.93 mmol) of LiCl was added. Stirring was kept at room temperature for an additional 2 h until a clear solution was obtained. Then, 24.28 mL (123.4 mmol) of TDMS-Cl were added to the gel, as well as 4.92 g (72.34 mmol) of imidazole. The reaction mixture was allowed to react at 100 °C for 24 h under stirring and later precipitated from an aqueous phosphate buffer solution. The precipitated polymer was recovered by filtration, washed with water and ethanol to remove excess silylating agent, and then dried at 60 °C under vacuum for 12 h. The obtained polymer was soluble in tetrahydrofuran, chloroform, and toluene. Physicochemical characterization of the obtained polymer (FTIR and ^1^H NMR are presented in the Supporting Information, Figure S1).

### 2.3 Coating Formulation and Electrode Fabrication With Protective Coating

Before the coating procedure, Li metal disks (Gelon, 200 μm thickness) were cut on a 14 mm diameter and polished mechanically until the removal of the native layer. The coating solution was achieved by mixing defined ratios of P(LiMTFSI) single-ion conducting polymer, cellulose material, and LiNO_3_ in anhydrous THF. The ratio selected in this study corresponds to a concentration of 5 μg cm^–2^ P(LiMTFSI), 100 μg cm^–2^ TDMSC, and 300 μg cm^–2^ LiNO_3_, depicted onward as a PTL protective layer. For the electrochemical study for optimal content selection, the reader is referred to Section S2 in the Supporting Information. The coating solution was prepared in an inert atmosphere, inside an Ar-filled glovebox (MBraun Unilab, H_2_O and O_2_ content < 1 ppm). The protective layer was formed using the drop-casting technique by applying 26.8 μL cm^–2^ of the coating solution to a polished Li metal electrode in three successive layers. THF was allowed to evaporate between each application, and the final coating was left to dry overnight (Figure 1b).

### 2.4 Physicochemical Characterization

The synthesized cellulose-based polymer was analyzed by infrared spectroscopy with a Bruker Alpha II equipped with a Ge ATR crystal. The spectra were collected in absorbance mode with 64 scans at a resolution of 4 cm^–1^ in the range of 4000 to 600 cm^–1^. ^1^H NMR spectrum was measured on a Bruker AVANCE NEO 600 MHz NMR spectrometer using CDCl_3_ solvent.

Field-emission scanning electron microscopy (FE-SEM, Supra 35 VP Carl Zeiss) equipped with an energy-dispersive X-ray spectrometer INCA Energy 400 (Oxford, UK) was used to obtain the top and cross-sectional morphological images of the coating. Samples were prepared in an Ar-filled glovebox and transferred in a custom-made vacuum transfer holder, which is opened in the SEM chamber under reduced pressure.

### 2.5 Battery Cell Fabrication and Electrochemical Testing

All laboratory-scale Li-metal test cells were assembled in an Ar-filled glovebox (O_2_/H_2_O < 0.1 ppm) and all electrochemical measurements were performed using a VMP3 Biologic potentiostat/galvanostat controlled by EC-Lab software. The electrochemical performance was evaluated in pouch cells with nickel contacts by stacking two lithium electrodes separated with a pressed separator (Celgard 2320, PP/PE/PP 20 μm, with an electrochemical active area of 1.13 cm^2^).^37^ The electrolyte quantity used in all cells was standardized to 17.7 μL cm^–2^ for both liquid electrolytes (1 M LiFSI in FEC:DEC 1:2 or 1 M LiFSI in DME) unless otherwise specified. Cells were rested for 20 h at OCV before cycling.

Li || Li symmetric cells (bare and coated) were subjected to galvanostatic cycling with a plating capacity of 2 mA h cm^–2^ at the current density of 1 mA cm^–2^. Li || Cu Coulombic efficiency (CE) tests were performed following a previously reported protocol,^38^ consisting of performing one full plating-stripping cycle at a capacity of 4 mA h cm^–2^ and current density of 0.4 mA cm^–2^, followed by a Li reservoir formation on the Cu electrode by applying 4 mA h cm^–2^ at the same current density. Later, the Li inventory was cycled 10 times with an areal capacity of 0.5 mA h cm^–2^ and current density of 0.4 mA cm^–2^, to finally complete an exhaustive Li stripping from the Cu electrode, applying 4 mA h cm^–2^ and 0.4 mA cm^–2^ and limiting the voltage to 1 V. The CE was calculated by comparing the capacity of the formed Li metal reservoir and the final stripping.

Full cell battery configuration for liquid electrolytes was evaluated by coupling coated and bare Li anodes with LiFePO_4_ (LFP) cathodes with a mass loading of approximately 6.3 mg cm^–2^ of active material. Composite cathodes were prepared by ball milling (300 rpm, 30 min) to properly mix active material (LFP), C65, and PVdF in a ratio of 90:5:5 wt %, using NMP as solvent. The prepared slurry was cast on carbon-coated aluminum foil using a doctor blade with a 200 μm gap and dried overnight at 100 °C under a vacuum. The cathodes were cut in 12 mm diameter and pressed at 2 ton cm^–2^ before being transferred to a glovebox. Li metal batteries were tested in a potential range between 2.5 and 4.1 V vs Li^0^/Li^+^ with three formation cycles at C/10 (16.97 mA g^–1^) and 1C (169.73 mA g^–1^) for the following cycles, by using 1 M LiFSI in DME as the electrolyte, with 100 μL soaked 16 mm diameter glassy fiber.

Solid-state battery cells were assembled using a self-standing solid polymer electrolyte (SPE), prepared by following a modified previous protocol.^39^ Briefly, LiTFSI and benzophenone (5%) were mixed at 60 °C in an Ar-filled glovebox, followed by the addition of PEO (EO:Li = 20:1) and hand-grinding with an agate mortar to form a paste-like consistency. Once the mixture is homogeneous, it was placed between two poly(ethylene terephthalate) (PET, Mylar) sheets and sealed inside a poly(propylene) (PP) bag to protect the materials from oxygen exposure during hot pressing. The resulting gum-like mixture was then hot-pressed at 75 °C for 15 min, applying 25 bar. Finally, the pressed film was UV-cured using a medium-pressure Hg lamp (Helios Quartz) with an intensity of 40 mW cm^–2^ and punched to the desired diameter inside the glovebox. The composite LFP-based cathode was formulated in a catholyte composition based on an adapted procedure,^40^ which in general involves the mixture of the active material (LFP), C65, PEO, and LiTFSI (EO:Li = 12:1) to form a slurry. 122.9 mg of LiTFSI was dissolved in 8 mL of acetonitrile and stirred for 10 min until total dissolution of the salt. Then, 226.9 mg of PEO was slowly added to the previous solution and stirred overnight. Later, 733 mg of LFP and 81.4 mg of C65 were transferred to a zirconia ball milling jar (25 mL capacity, with 20 zirconia balls of 14 mm diameter), and the mixture of PEO-LiTFSI in acetonitrile was added to achieve a weight ratio of 63:7:19.4:10.6. Finally, the mixture was ball milled with 0.5 mL of NMP to complete 4 cycles of 30 min at 300 rpm +10 min interval rest at room temperature. Afterward, the slurry was cast on a C-coated aluminum current collector using a doctor blade gap of 200 μm and then dried at room temperature for 5 h. The resulting catholyte was vacuum-dried at 50 °C overnight, followed by a densification process using a hot rolling machine (HR01 MTI) with a gap distance of 200 μm at 50 °C. Once the process was completed, the electrodes were cut into 12 mm diameter disks and dried under vacuum at 50 °C before being placed in the glovebox. The cathode active material mass loading was around 2 mg cm^–2^ for coated and bare Li metal cells. The solid-state cells were prepared in pouch cells with nickel contacts by stacking a lithium metal anode and the LFP catholyte with the PEO-based SPE in between. The electrochemical behavior was recorded at 60 °C after resting 12 h at OCV, in a potential range between 2.5 and 4.1 V vs Li^0^/Li^+^, at different C-rates of C/10, C/5, C/2, and 1C (169.19 mA g^–1^).

## 3 Results and Discussion

### 3.1 PLiMTFSI-TDMS-LiNO_3_ Protective Coating on Li-Metal (Li@PTL)

**Figure 2:**
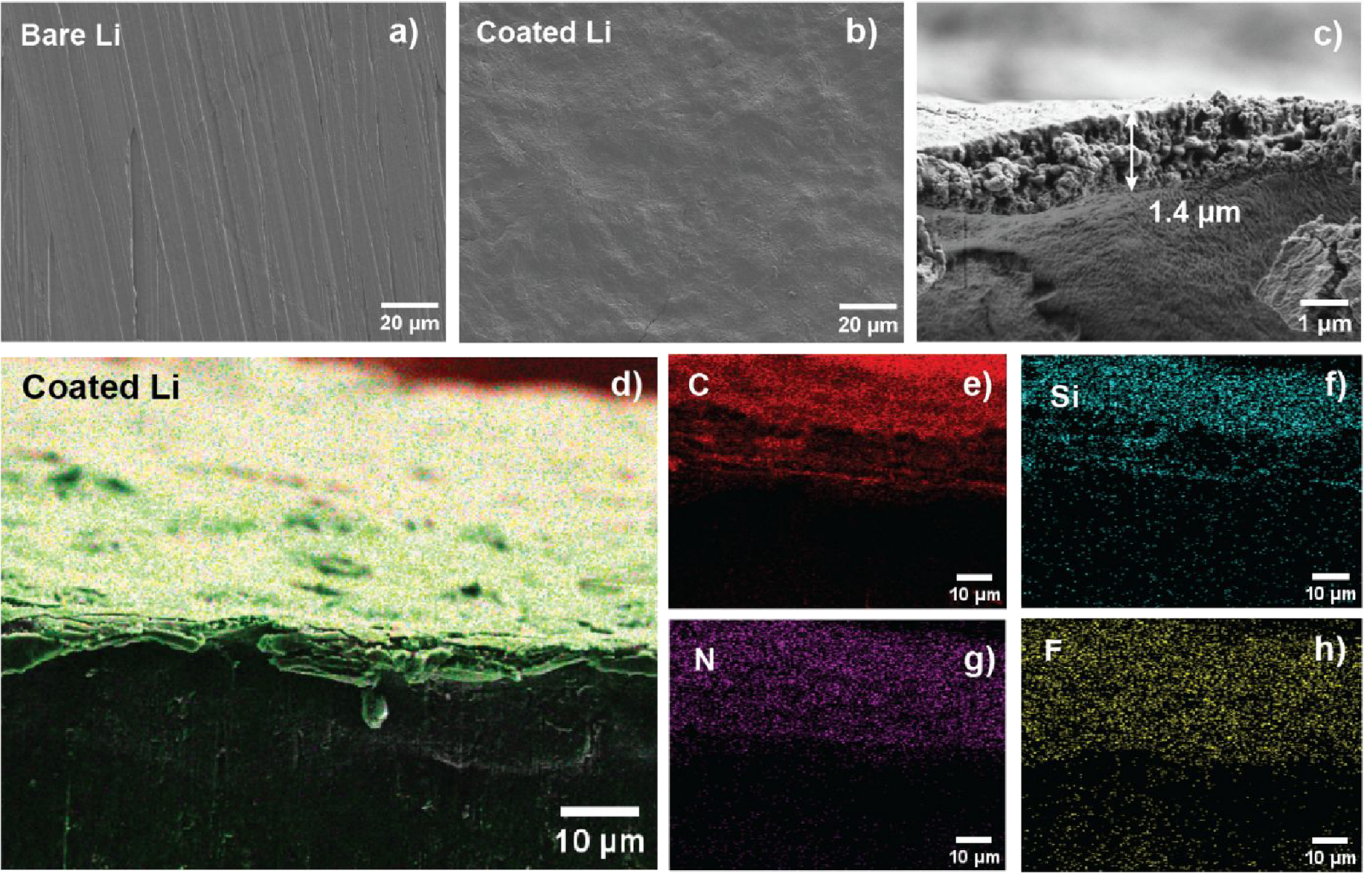
SEM micrographs of (a) bare Li metal, (b) coated Li metal with 5 μg cm^–2^ P(LiMTFSI), 100 μg cm^–2^ TDMSC, and 300 μg cm^–2^ LiNO_3_, Li@PTL, and (c) cross section of coating applied on Li metal. (d) EDX mapping of coated Li@PTL cross-section showing the elemental distribution of (e) carbon, (f) silicon, (g) nitrogen, and (h) fluorine.

The optimization of P(LiMTFSI) content in the protective coating evaluated by galvanostatic cycling in symmetrical Li || Li and asymmetrical Li || Cu cells in 1 M LiFSI in FEC:DEC is presented in Figure S2. Additionally, we analyzed the morphology of the metallic lithium surface with FE-SEM, both before and after applying the PTL protective coating (5 P(LiMTFSI)-100 TDMSC). In the pristine (bare) Li metal, we observed continuous groove formation with a special hatch distance of about 5 μm caused by mechanical surface activation (Figure 2a). In contrast, the PTL-coated Li metal (Figure 2b) showed a homogeneous and smooth surface, with the protective layer conformally adapting to the pristine Li metal roughness. The thickness of the protective layer was obtained from the SEM cross-section by previously freezing the sample (inside a Triplex pouch bag) by immersion in liquid nitrogen, resulting in a range of 1.4 to 1.6 μm (Figure 2c). Due to its thickness and formation method, the coating obtained is not self-standing, drop-casting assures an optimal contact between Li metal by directly laminating the new interface to achieve the thinnest possible coating.

Energy dispersive X-ray (EDX) mapping (Figure 2d) shows a uniform distribution of carbon, silicon, nitrogen, and fluorine, confirming the homogeneous distribution of the three PTL components, P(LiMTFSI), TDMSC, and LiNO_3_. However, the multiple sources of carbon, silicon, nitrogen, and fluorine (Figure 2e-h) limit reliable calculation of the atomic ratio between species.

### 3.2 Electrochemical Behavior in Li–Li Symmetrical Cells

**Figure 3:**
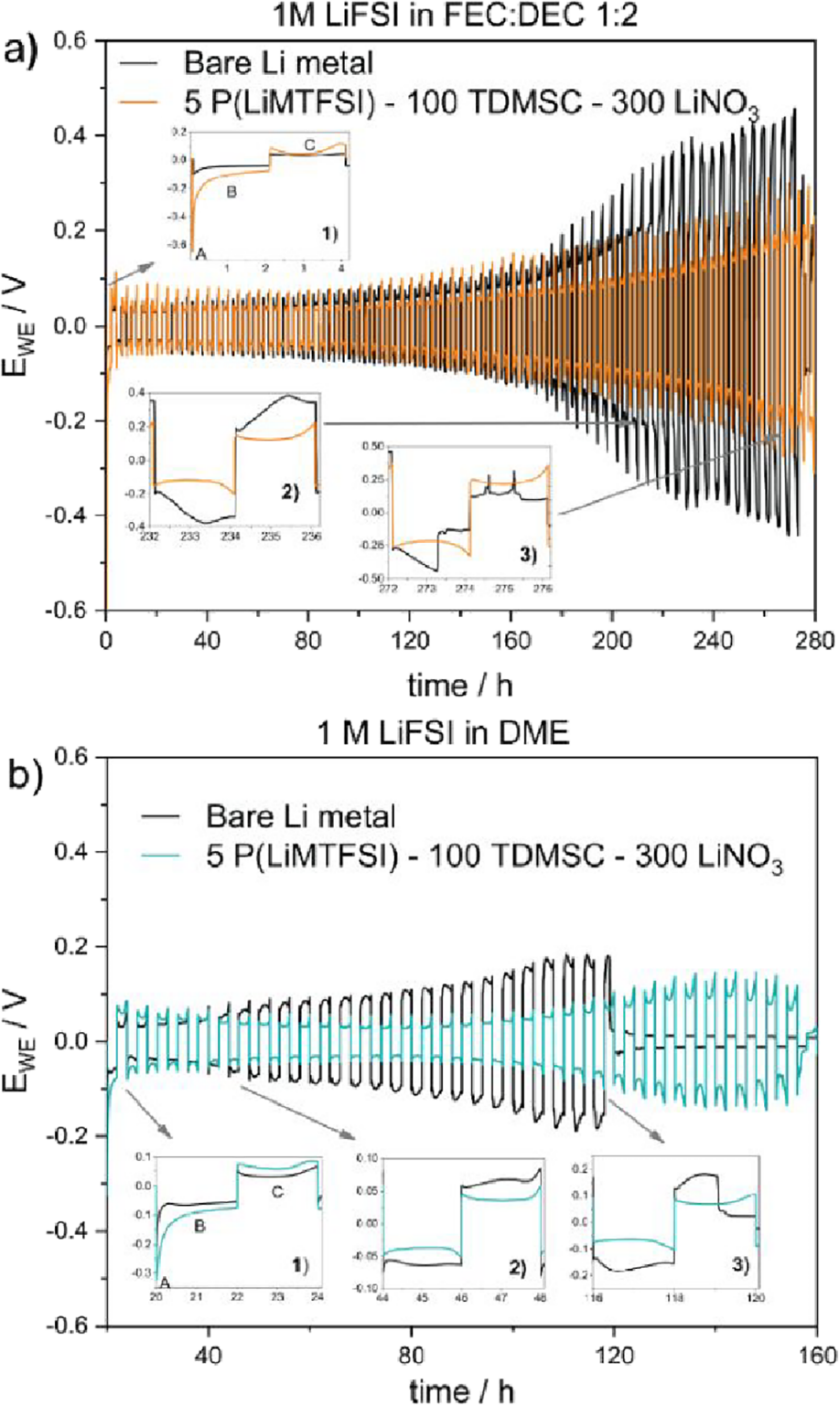
Galvanostatic cycling of bare and coated Li || Li symmetric cells at areal capacity of 2 mA h cm^–2^ with a current density of 1 mA cm^–2^ in (a) 1 M LiFSI in FEC:DEC 1:2 and (b) 1 M LiFSI in DME, displaying the characteristic galvanostatic traces over time.

We analyzed the electrochemical performance of Li metal electrodes in both symmetric and asymmetric cells to obtain complementary information. In the first setup, Li || Li symmetric cells were used, where the electrodes underwent continuous plating and stripping at an areal capacity of 2 mA h cm^–2^ and a current density of 1 mA cm^–2^ per charge/discharge cycle. This approach allowed us to track overpotential changes during cycling, providing information about mass transport at the electrolyte-Li metal interface and evaluating coating stability.^41^ In cells tested with 1 M LiFSI in an FEC:DEC electrolyte, bare Li metal exhibited an initial nucleation overpotential of 100 mV (Figure 3a, inset 1, region A). In contrast, the coated Li || Li cell showed an overpotential of 600 mV, indicating the additional interphase created by the protective coating (Figure 3a).

After nucleation, a steady plateau is reached, reflecting a rapid equilibrium where Li^+^ concentration gradients are negligible (Figure 3a, inset 1, region B), producing the characteristic “peaking” voltage trace visible at the edges of region C. After 230 h of cycling, evident changes are observed in the bare Li metal cell, where a transition from “arc” to “peak” indicated the buildup of electrically disconnected, or “dead,” lithium.^42^ The “arc” shape arises from increased tortuosity at the electrode surface, which elongates the pathway that Li^+^ has to travel to reach the metallic surface, creating a mass transport limitation (Figure 3a, inset 2). Ultimately, the bare Li electrode shows clear signs of short-circuiting due to high-surface-area lithium (HSAL) generation (Figure 3a, inset 3). In contrast, Li@PTL cells cycle with a 30% lower overpotential throughout testing, avoiding the previously observed “arching” behavior or clear signs of short circuits.

We noticed a similar behavior in DME-based electrolytes (Figure 3b inset 1); however, the bare Li metal electrode shows the transition from peak to arc trace as early as 44 h of cycling followed by the cell failure (Figure 3b insets 2 and 3). Also, in the Li@PTL, the overpotential decreased from 70 to 40 mV after 50 h, showing the creation of conduction pathways through the protective coating. The cycling life of the PTL-coated lithium metal cell was extended by around three times (considering the beginning of the arching transition in the bare Li metal cell) demonstrating that the presence of the protective layer improves the stability of the cell as well as inhibits HSAL production.

### 3.3 Electrochemical Behavior in Li–Cu Asymmetrical Cells

**Figure 4:**
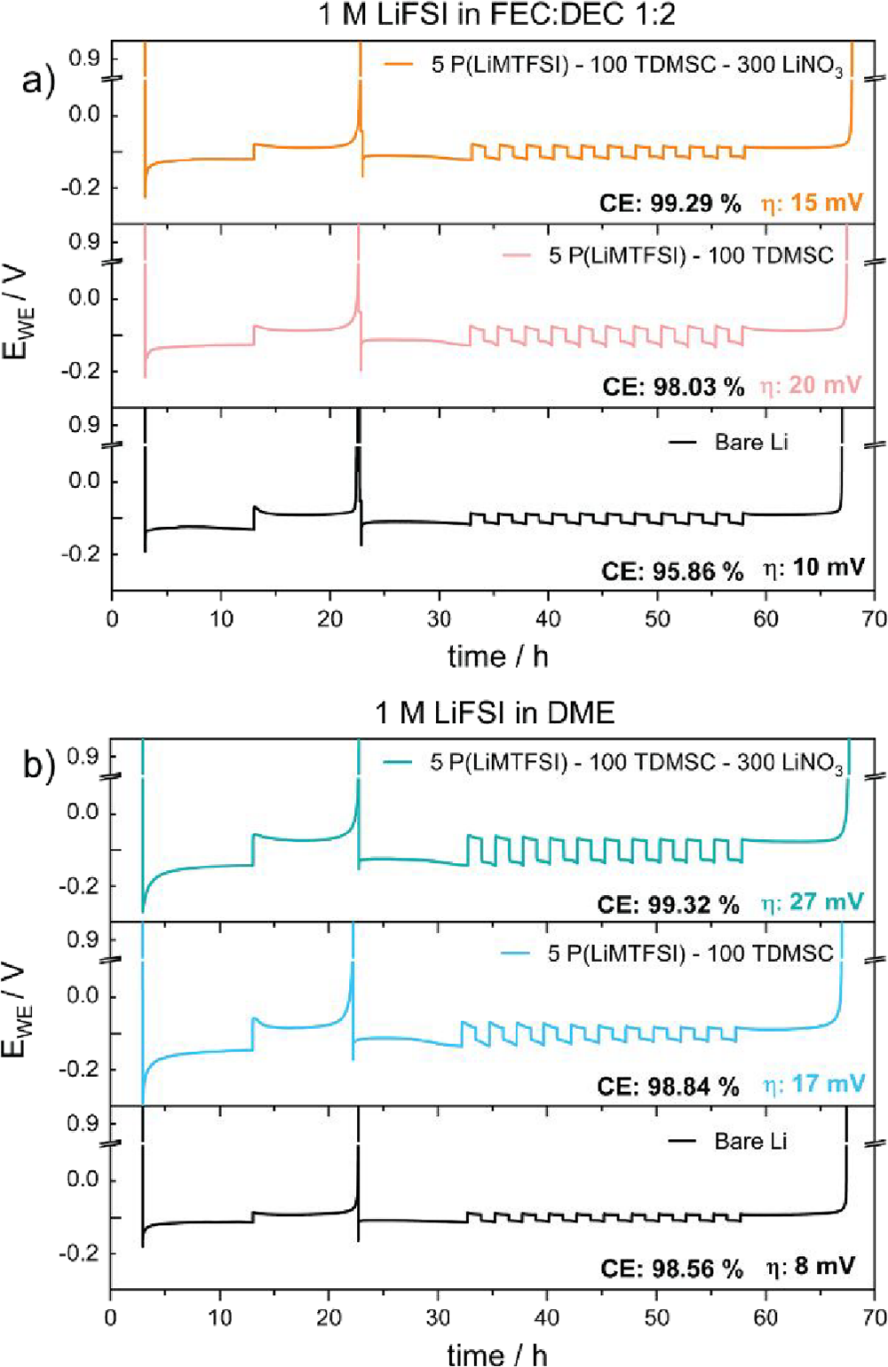
Utilization tests using Adams protocol^38^ to calculate Coulombic efficiency of bare, TDMSC + P(LiMTFSI), and TDMSC + P(LiMTFSI) + LiNO_3_ coated Li metal (Li@PTL) in (a) 1 M LiFSI in FEC:DEC and (b) 1 M LiFSI in DME electrolyte.

In symmetric Li || Li cells, the unlimited supply of Li makes it impossible to accurately quantify the Coulombic efficiency (CE). To address this, we used asymmetric Li|Cu cells with a finite amount of Li. In this setup, a fixed amount of Li (17.2 μm, equivalent to a capacity of 4 mA h cm^–2^) was plated onto the Cu electrode. The cell was then cycled 10 times with 0.5 mA h cm^–2^ per cycle (corresponding to 2.16 μm of lithium plated and stripped) before an exhaustive final stripping of Cu at 4 mA h cm^–2^. This approach allows us to accurately calculate the CE by comparing the charge from the initial Li reservoir to the final stripping charge.^38^ In addition, the use of high-capacity preconditioning stabilizes the copper substrate, which reduces the effects of passivation that can lead to uncertainties in the CE calculation (“ramp up” effect).^38^Figure 4 shows the evaluation of the Li || Cu asymmetric cells for bare, TDMSC + P(LiMTFSI) coating and Li@PTL configurations, both in carbonate (Figure 4a) and ether (Figure 4b) electrolytes. Similar to the case for the symmetrical cells, the increase in overpotential can be related to the presence of the protective layer on the Li metal surface. However, with the addition of LiNO_3_, we notice a 5 mV decrease in the overpotential (compared to the TDMSC + P(LiMTFSI) coating) as well as a flatter galvanostatic curve with a lower nucleation energy barrier and improved kinetics. The utilization of the Li metal also increases from 95.9% for the bare Li metal electrode to 98.0% for the TDMSC + P(LiMTFSI) coated Li metal electrode. A CE of 99.3% was achieved by adding LiNO_3_ to the polymer matrix, which could be explained by an increase in ionic conductivity as well as the blocking of porosity in the polymer matrix. The impact of the coating on Li morphology is shown in the Supporting Information, Figure S3, where Li deposits are observed in rounded shapes and compact films (Figure S3c). These results prove that the addition of the composite coating increases the utilization efficiency and, thus, the prospects of Li-metal battery application. The differences in overpotential between the carbonate- and ether-based electrolytes are likely related to the different transport mechanisms and the interaction between electrolyte and coating components, but a full discussion is beyond the scope of this work and will be addressed in a follow-up work.

### 3.4 Li Metal Battery Cell Testing in Liquid and Solid-State Electrolyte Configurations

#### 3.4.1 Li || LFP Cell with Liquid Electrolyte

**Figure 5:**
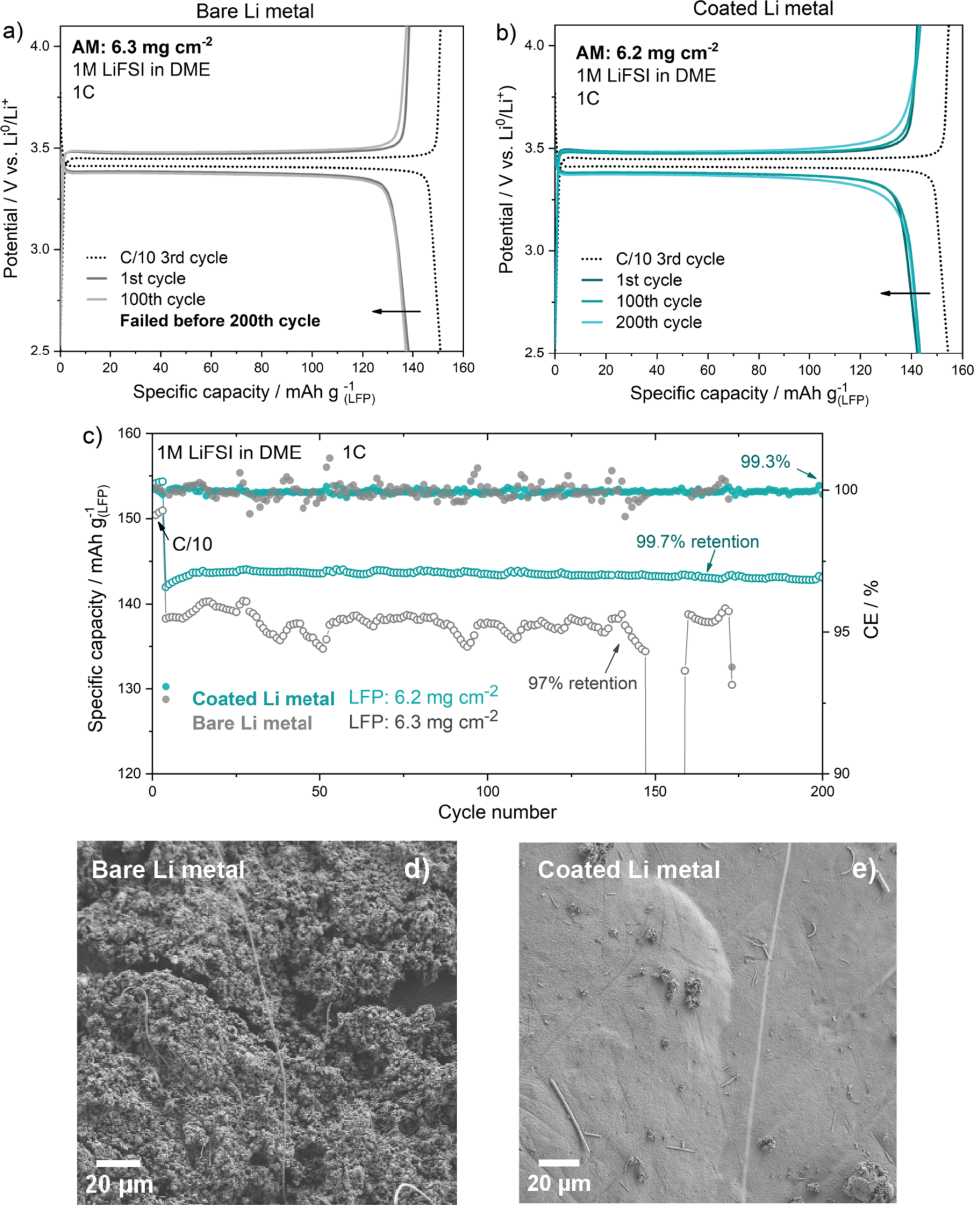
Galvanostatic charge/discharge profiles of Li || LFP battery cells assembled with (a) bare Li metal and (b) Li@PTL anodes, using 1 M LiFSI in DME as the electrolyte, at 1C rate. (c) Related capacity retention and CE during prolonged cycling, up to 200 cycles at 1C. SEM micrographs of (d) bare Li metal and (e) Li@PTL electrodes after 50 charge/discharge cycles at 1C.

To demonstrate the applicability of the PTL coating under study, Li || LFP batteries were prepared by coupling bare and coated Li metal with LFP cathodes, having a mass loading of 6.3 and 6.2 mg cm^–2^ of active material, which corresponds to an areal capacity of 1.1 and 1.06 mA h cm^–2^, respectively. The resulting cells were tested with an ether-based electrolyte (1 M LiFSI DME). Figure 5a,b shows the charge–discharge profiles of LFP cycled with bare and Li@PTL. The cell was precycled at C/10 (3 initial cycles) and then continuously cycled at 1C. During the formation cycles, specific capacity values of 151 and 155 mA h g^–1^ were obtained for bare and Li@PTL, which corresponds to 88.8 and 91.2%, of LFP theoretical capacity (170 mA h g^–1^).

The specific capacity provided by the bare Li cell at 1C is 138 mA h g^–1^ with notable fluctuations, and before completing 150 cycles, the reversible capacity drops drastically leading to failure (Figure 5c). CE values above 100% are noticed, which are commonly related to side reactions at the anodic side and HSAL growth and propagation. Conversely, the cell assembled with Li@PTL anode exhibits a stable specific capacity of 143 mA h g^–1^ over 200 cycles with 99.3% CE, and almost no capacity fading, demonstrating the coating’s ability to prevent the continuous degradation of Li metal anode. To prove this, FE-SEM was used to observe the electrode surface morphology after cycling. Therefore, both cells were stopped after 50 cycles, and Li metal electrodes were recovered, washed with fresh DME, and vacuum-dried overnight. The bare Li electrode shows the typical porous high surface area + dead lithium structure, that covers almost the totality of the electrode surface (Figure 5d). On the contrary, the Li@PTL metal anode shows a rather smooth surface (Figure 5e); although some particles and surface formations are present, the coating appears to be still homogeneous and intact.

#### 3.4.2 Li || LFP Cell with Solid-State Electrolyte

**Figure 6:**
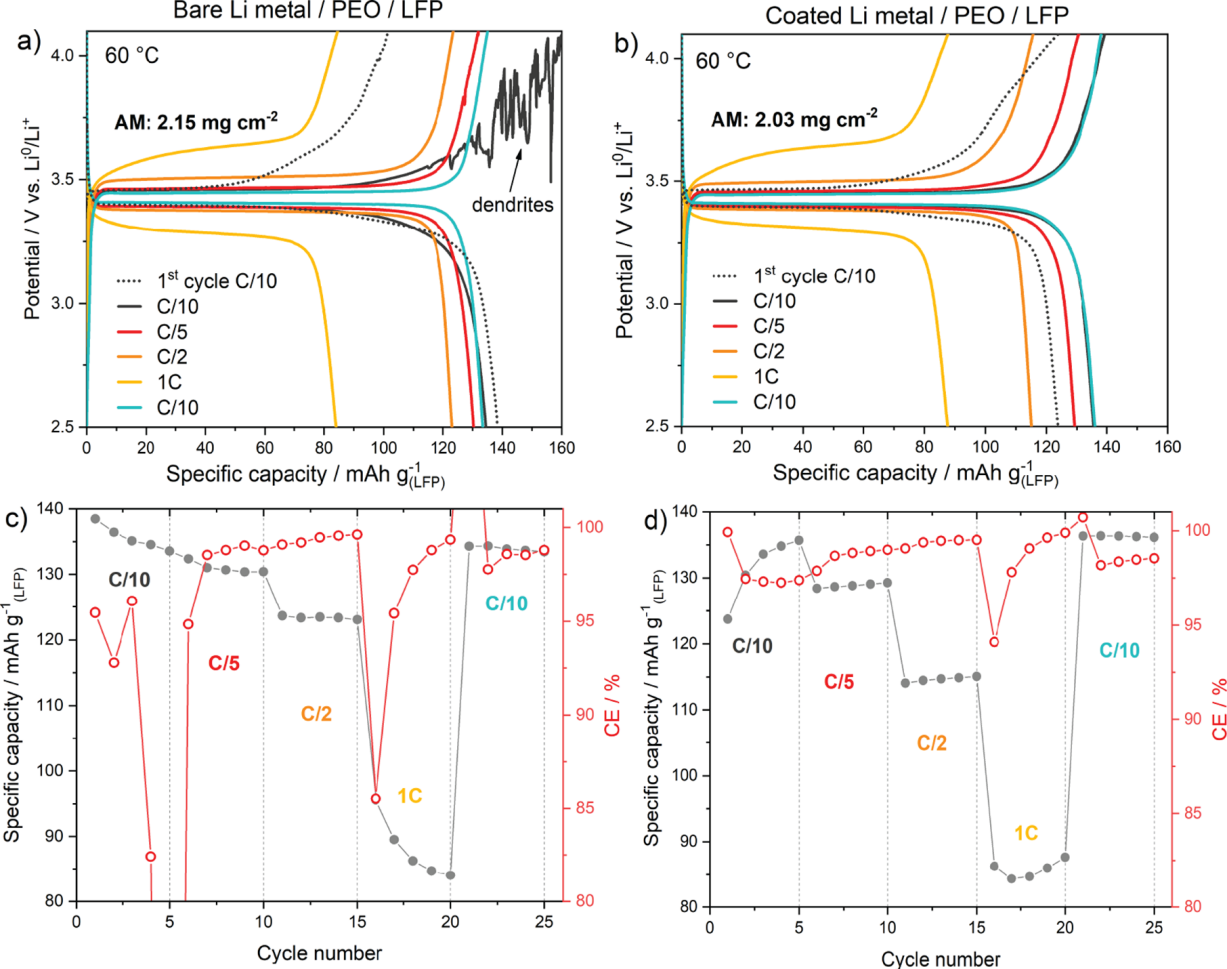
(a, b) Galvanostatic cycling behavior of bare Li || LFP and Li-coated PTL || LFP solid-state cell at 60 °C during the rate capability test and (c, d) corresponding capacity retention vs CE plot at different constant current rates.

As a proof of concept to explore further the promising prospects for application of the PTL-based coating under study, we tested it in an all-solid-state battery cell configuration, which was assembled by combining a Li@PTL anode with an LFP-based catholyte containing 2.03 mg cm^–2^ of active material. The rate capability test was performed at 60 °C, and the current rate was continuously increased from C/10 to 1C (0.34 mAh cm^–2^) in a potential window of 2.5 to 4.1 V a. Figure 6a,b shows the galvanostatic charge/discharge profiles at different C-rates, for both bare and coated Li anode, where the plateau indicates the characteristic LFP redox reactions. The appearance of Li dendrites is evident in bare Li cells, Figure 6a,c, starting from voltages close to 3.6 V, which is also reflected in abrupt changes in Coulombic efficiency and fluctuating specific capacity. PEO decomposition starts at 3.8 V, further aggravated by temperature, accelerating capacity fading.^43^ Which can be the reason for efficiencies lower than 100%. In contrast, coated Li metal provides smooth charge/discharge cycles, with an initial activation at C/10, where the specific capacity increases as the contact between the cell components improves, Figure 6b,d.

**Figure 7:**
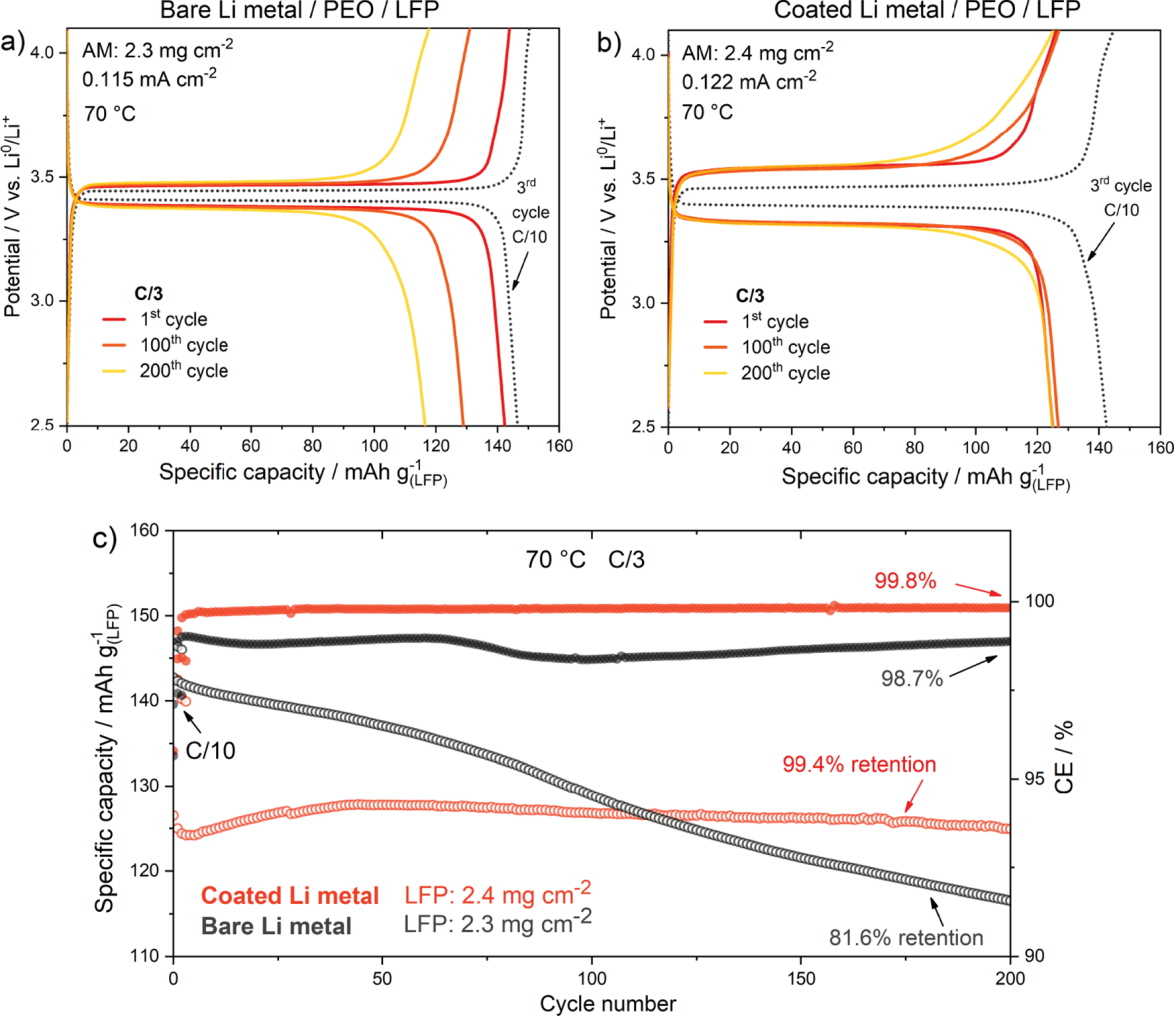
(a, b) Long-term galvanostatic charge/discharge of bare Li || LFP and Li-coated || LFP solid-state cell at 70 °C and at C/3. (c) Related specific capacity retention and CE during prolonged cycling, 200 cycles at C/3.

The solid-state coated Li-metal cell provides a specific capacity of 135 mA h g^–1^ at C/10, which gradually decreases to 87 mA h g^–1^ at 1C, accounting for a rather good capacity retention considering the solid-state cell configuration. Remarkably, in Figure 6b, the coated Li cell fully recovers the specific capacity when the current rate is reduced back to C/10. It suggests the structural stability of the protective coating onto the Li metal anode and its beneficial effects on the interfacial properties. Additionally, we observed a constant CE exceeding 98% (Figure 6d), which accounts for the positive influence of the PTL protective coating on the interfacial properties by also improving the adhesion with the Li metal (Supporting Information S4). In solid-state batteries, the ionic conductivity is controlled by the contact area at the interfaces between the SPE and electrodes. These results suggest that the presence of the coating improves the interfacial contact of the SPE at the Li metal surface and enables the creation of Li^+^ conduction pathways, which is reflected in a more homogeneous Li^+^ transport, and increased stability between Li metal and the SPE. Additionally, to demonstrate the coating’s stability, we conducted a long-term cycling test on solid-state configuration, Figure 7. The Li-coated cell maintained a capacity of 128 mA h g^–1^ at C/3, for 200 h, shown in Figure 7b,c, having an overall specific capacity retention of 99.4% and a CE of 99.8%. In contrast, the bare-Li cell (Figure 7a,c) showed a declining specific capacity, retaining only 81.6% and a CE of 98.7%, highlighting the stabilizing effect of the PTL protective coating.

## Conclusions

In this work, we have shown that the synergy between a cellulose-based polymer (TDMSC), a single-ion conducting polymer (PLiMTFSI), and LiNO_3_ enables the creation of a stable Li-metal protective coating that extends the cycle life and mitigates the effects of high surface area lithium (HSAL). The performance of the protective coating was evaluated in both symmetrical Li || Li and asymmetrical Li || Cu cells to assess stability and Coulombic efficiency, respectively. Li || Li PTL-coated cells showed a longer, more stable cycle life and a slower increase in overpotential compared to the bare Li counterpart at a current density of 1 mA cm^–2^ and a capacity of 2 mA h cm^–2^. Li || Cu showed an improved CE exceeding 99.3% in both carbonate- and ether-based electrolytes. In contrast, cells assembled with bare Li showed reduced CE values of 95.9 and 98.6% in carbonate- and ether-based electrolytes, respectively. Laboratory-scale Li metal pouch cells were assembled to test the coating with one of the most commercially relevant cathodes (LFP), resulting in increased specific capacity and Coulombic efficiency without the generation of HSAL in liquid electrolyte, which was confirmed by postcycling FE-SEM analysis. In addition, the PTL protective layer promoted dendrite-free cycling in solid-state battery cells assembled with LFP in a catholyte configuration (PEO + LiTFSI as an ionically conductive binder) and a PEO-based cross-linked solid polymer electrolyte. Galvanostatic cycling reflected an improved Li^+^ transport at the electrode–electrolyte interface, which allowed for higher stability between Li metal and SPE upon prolonged operation. Thus, we demonstrated the role of interface stabilization as well as the protective and conductive nature of the coating during cycling, forming homogeneous lithium plating and accounting for its promising prospects for the future development of stable and safe Li metal batteries.

## Supporting Information Available

Physicochemical characterization of TDMSC material (^1^H NMR spectra and FTIR), optimization of TDMSC-P(LiMTFSI) coating formulation by Li || Li and Li || Cu, morphology of Li plated on coated and bare Cu at 0.5 mA h cm^–2^, and cross section of Li/LFP/Li cell configuration (PDF)
