# Supplementary material for: Protective Coating for Stable Cycling of Li-Metal Batteries Based on Cellulose and Single-Ion Conducting Polymer
Source: ACS Appl Mater Interfaces. 2024 Nov 25;16(49):68237–46. doi: 10.1021/acsami.4c13335 (PMC11647898; doi:10.1021/acsami.4c13335)
Supplement: Supplementary file 1 — am4c13335_si_001.pdf [file am4c13335_si_001.pdf]

SUPPORTING INFORMATION

Protective Coating for Stable Cycling of Li-Metal Batteries Based on Cellulose and Single-Ion Conducting Polymer

Mariana Vargas Ordaz [a, b, d], Nejc Pavlin [a], Matteo Gastaldi [c], Claudio Gerbaldi [c, e], Robert Dominko [a, b, d]*

[a] National Institute of Chemistry, Hajdrihova 19, SI-1000, Ljubljana, Slovenia

[b] University of Ljubljana, Faculty of Chemistry and Chemical Technology, Večna pot 113, SI-1001, Ljubljana, Slovenia

[c] GAME Lab, Department of Applied Science and Technology (DISAT), Politecnico di Torino, Corso Duca degli Abruzzi 24, 10129, Torino, Italy

[d] ALISTORE -European Research Institute, 33 rue Saint-Leu, Amiens 80039 Cedex, France

[e] National Reference Center for Electrochemical Energy Storage (GISEL) – INSTM, Via G. Giusti 9, 50121, Firenze, Italy

*Corresponding author.

E-mail: robert.dominko@ki.si

S1. Synthesis and characterization of 2-6-di-O-thexyldimethylsilylcellulose

TDMSC was obtained from the silylation of cellulose with thexyldimethylchlorosilane (TDMS-Cl) in *N*,*N*-dimethylacetamide (DMAc)/LiCl solution in the presence of imidazole. After 24 h at 100 °C the reaction became heterogeneous (particles in suspension were observed in the media), and the product was precipitated from a phosphate buffer solution. The polymer was washed thoroughly with water and ethanol to remove the excess of silylating agent, to obtain a white powder. The structure of 2-6-di-O-thexyldimethylsilylcellulose was confirmed by FTIR, where the characteristic bands were observed at 3500 cm^{-1} (weak, -OH stretching vibration), 2969, 2867 cm^{-1} (-CH asymmetric and symmetric stretching vibrations, respectively), 1466 cm^{-1} (-CH, $-CH_2$ bending), 1252, 833, 778 cm^{-1} (SiC), 1152, 1119, 1078, 1038 cm^{-1} (C-O-C stretching vibration of cellulose anhydroglucose unit (AGU)),[1,2] proving the successful conversion of cellulose to TDMSC, Fig 1a). In cellulose, the maximum substitution degree is 3, representing the total reaction of the three hydroxyl groups.[3] The degree of substitution (DS) of TDMSC was calculated from the ^1H NMR spectra resulting in 2.6. The methodology followed was described elsewhere,[4] which in our case, the integral for protons linked to Si moiety (0.2 to -0.15 ppm) is calculated based on setting the cellulose backbone of an AGU integral to 7 H, (4.5 to 3.0 ppm), Fig 1b).

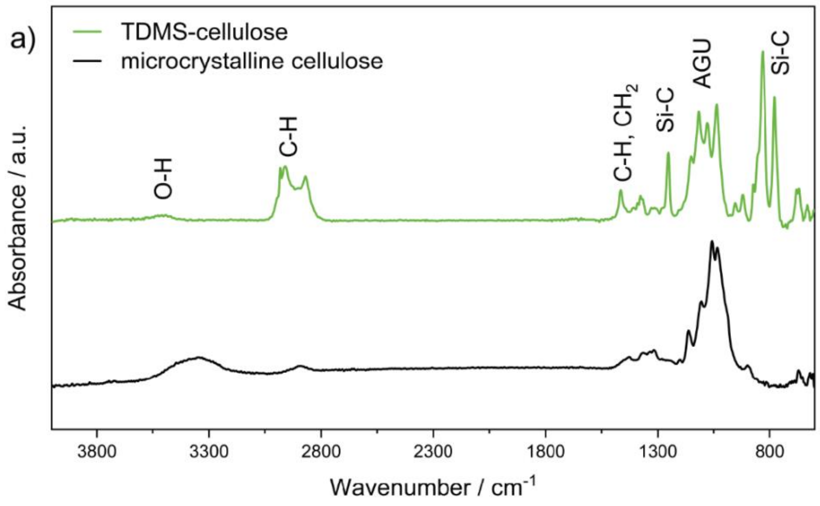

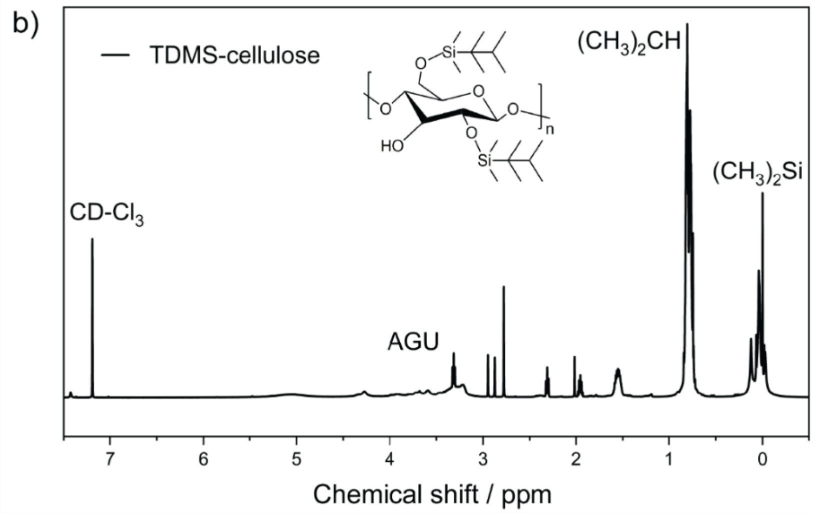

Figure S1. a) FTIR of TDMSC using Ge crystal, and b) ^{1}H NMR spectra of TDMSC in $CDCl_3$.

S2. Optimization of single-ion conducting polymer content for coating formulation

We selected 100 µg cm^{-2} of TDMSC as the ideal cellulose content to further tune the P(LiMTFSI) concentration and obtain the desired electrochemical performance for the polymeric matrix. 1, 5, and 10 µg cm^{-2} of P(LiMTFSI) were tested on the basis of Li||Li (plating capacity of 2 mAh cm^{-2} at 1 mA cm^{-2}), and Li||Cu cell configuration, following a previously described protocol,[5] including a copper pre-treatment at a capacity of 4 mAh cm^{-2} at 0.4 mA cm^{-2}, followed by a Li reservoir formation on the Cu electrode with 4 mAh cm^{-2} at the same current density. Later, the Li inventory was cycled 10 times at 0.5 mAh cm^{-2} (0.4 mA cm^{-2}), to finally strip Li from the Cu electrode, applying 4 mAh cm^{-2} at 0.4 mA cm^{-2} limiting the voltage to 1 V. The CE was calculated by comparing the capacity of the formed Li metal reservoir and the final stripping.

Based on these results, 5 µg cm^{-2} of P(LiMTFSI) was selected as the most suitable areal loading, by having the best trade-off between long-term stability and CE% (98.03%) that was further improved with the addition of $LiNO_3$.

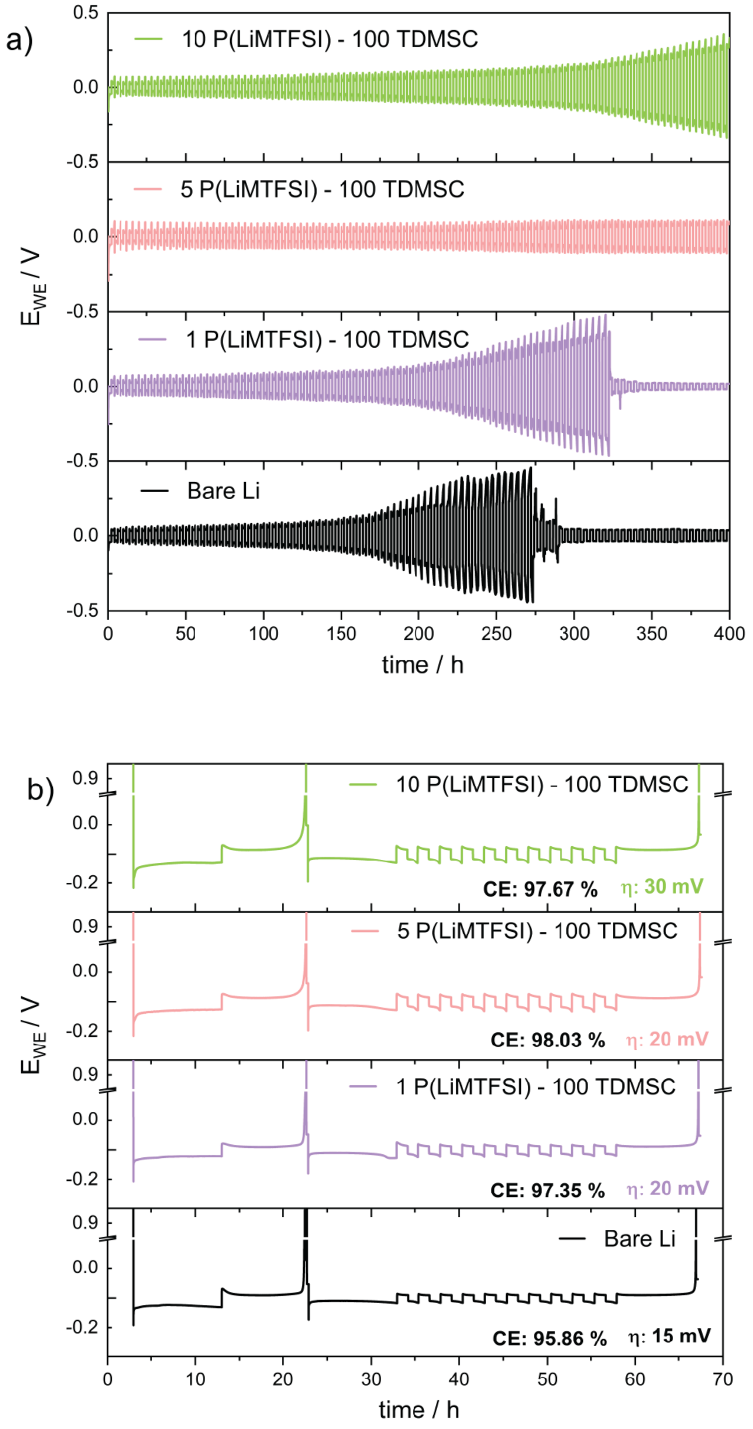

Figure S2. Optimization of P(LiMTFSI) content evaluated by galvanostatic cycling in a) symmetrical Li||Li and b) asymmetrical Li||Cu cells in 1M LiFSI in FEC:DEC.

S3. Morphology of Li plated on coated and bare Cu at 0.5 mAh cm^{-2}

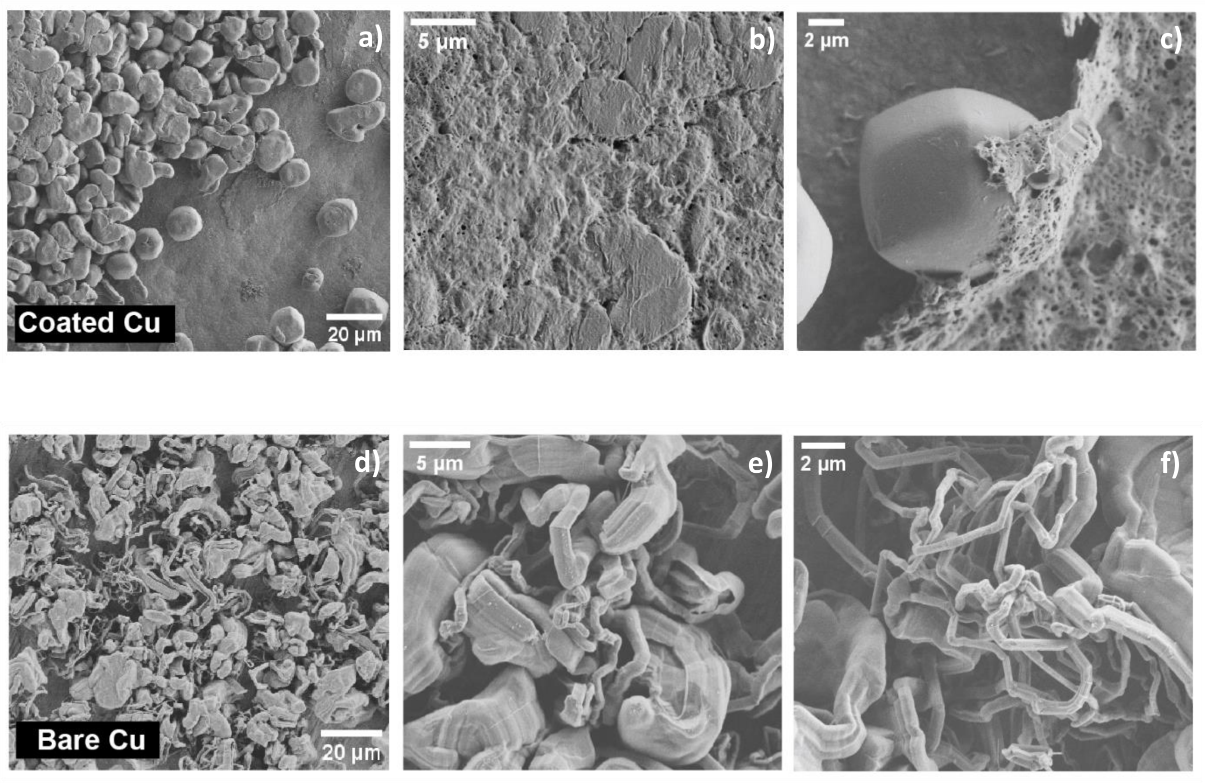

Figure S3. Li plated on coated and bare Cu at a plating capacity of 0.5 mAh cm^{-2}. Electrolyte 1M LiFSI in DME.

The Li deposits exhibit a spherical morphology, adapting to the shape of the coating in a compact and uniform manner. These spherical Li particles appear to be well-adhered to the coating surface, indicating strong interfacial contact between the deposited Li and the protective layer. In contrast, we observe the dendritic growth that Li typically presents on bare Cu electrode.

S4. Cross-section of Li/SPE/Li cell configuration

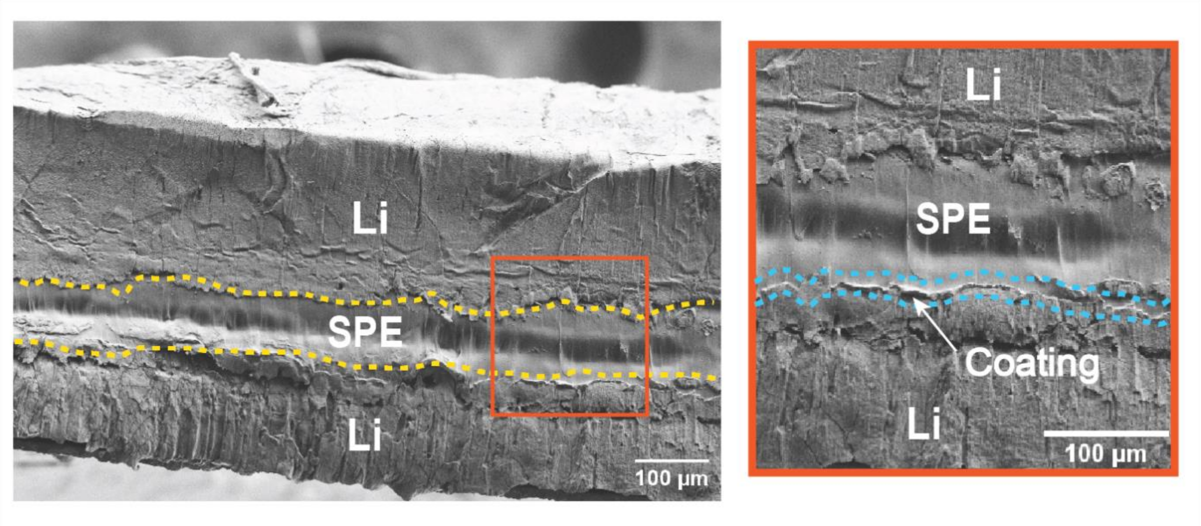

Figure S4. SEM cross-section micrograph of TDMSC-P(LiMTFSI)-$LiNO_3$-coated Li metal symmetric cell in a solid-state configuration. SPE = crosslinked PEO:LiTFSI (EO:Li =20:1).

REFERENCES

(1) Koschella, A.; Heinze, T.; Klemm, D. First Synthesis of 3-O-Functionalized Cellulose Ethers via 2,6-Di-O-Protected Silyl Cellulose; Macromol. Biosci. 2001, 1 (1), 49-54. https://doi.org/https://doi.org/10.1002/1616-5195(200101)1:1<49::AID-MABI49>3.0.CO;2-C.

(2) Infrared Analysis of Organosilicon Compounds: Spectra-Structure Correlations. Complied by Philip J. Launer, Updated by Barry Arkles. Reprinted from Silicon Compounds: Silanes and Silicones, 2013 Gelest, Inc Morrisville, PA. https://www.gelest.com/wp-content/uploads/5000A_Section1_InfraredAnalysis.pdf (accessed 2024-08-01)

(3) Jett C. Arthur Jr. Chemical Modification of Cellulose and Its Derivates. Comprehensive Polymer Science and Supplements; 1989, 6, 49–80. https://doi.org/https://doi.org/10.1016/B978-0-08-096701-1.00182-8.

(4) Lindemann, H.; Heinze, Th. Cellulose Allylcarbamate with High Content of Reactive Double Bonds for Thiol-Ene Reaction. React. Funct. Polym. 2022, 176, 105306. https://doi.org/10.1016/j.reactfunctpolym.2022.105306.

(5) Adams, B. D.; Zheng, J.; Ren, X.; Xu, W.; Zhang, J. G. Accurate Determination of Coulombic Efficiency for Lithium Metal Anodes and Lithium Metal Batteries. Adv. Energy Mater. 2018, 8 (7). https://doi.org/10.1002/aenm.201702097.
